# Comparing immunogenicity and efficacy of two different mRNA-based COVID-19 vaccines as a fourth dose; six-month follow-up, Israel, 27 December 2021 to 24 July 2022

**DOI:** 10.2807/1560-7917.ES.2022.27.39.2200701

**Published:** 2022-09-29

**Authors:** Noam Barda, Michal Canetti, Mayan Gilboa, Victoria Indenboim, Keren Asraf, Yael Weiss-Ottolenghi, Sharon Amit, Daniel Zibly, Ram Doolman, Ella Mendelson, Dror Harats, Laurence S. Freedman, Yitshak Kreiss, Yaniv Lustig, Gili Regev-Yochay

**Affiliations:** 1 ARC Innovation Center, Sheba Medical Center, Tel Hashomer, Ramat Gan, Israel; 2 Software and Information Systems Engineering, Ben-Gurion University of the Negev, Be’er Sheva, Israel; 3 Epidemiology, Biostatistics and Community Health Services, Ben-Gurion University of the Negev, Be’er Sheva, Israel; 4 The Infection Prevention & Control Unit, Sheba Medical Center, Tel Hashomer, Ramat Gan, Israel; 5 Sackler School of Medicine, Tel-Aviv University, Tel Aviv, Israel; 6 Central Virology Laboratory, Public Health Services, Ministry of Health, Tel-Hashomer, Ramat Gan, Israel; 7 The Dworman Automated-Mega Laboratory, Sheba Medical Center, Tel-Hashomer, Ramat-Gan, Israel; 8 Clinical Microbiology, Sheba Medical Center, Tel Hashomer, Ramat Gan, Israel; 9 General Management, Sheba Medical Center, Tel Hashomer, Ramat Gan, Israel; 10 Biostatistics and Biomathematics Unit, Gertner Institute of Epidemiology and Health Policy Research, Sheba Medical Center, Tel Hashomer, Israel

**Keywords:** COVID-19, boosters, mRNA1273, BNT162b2, immunogenicity, vaccine efficacy

## Abstract

We assess the immunogenicity and efficacy of Spikevax and Comirnaty as fourth dose COVID-19 vaccines. Six months post-fourth-dose, IgG levels were higher than pre-fourth dose at 1.58-fold (95% CI: 1.27–1.97) in Spikevax and 1.16-fold (95% CI: 0.98–1.37) in Comirnaty vaccinees. Nearly 60% (159/274) of vaccinees contracted SARS-CoV-2. Infection hazard ratios (HRs) for Spikevax (0.82; 95% CI: 0.62–1.09) and Comirnaty (0.86; 95% CI: 0.65–1.13) vaccinees were similar, as were substantial-disease HRs, i.e. 0.28 (95% CI: 0.13–0.62) and 0.51 (95% CI: 0.27–0.96), respectively.

From two mRNA vaccines currently available, Comirnaty (BNT162b2, BioNTech-Pfizer, Mainz, Germany/New York, United States (US)) [1] and Spikevax (mRNA-1273, Moderna, Cambridge, US) [2], it is unknown which one leads to better long-term immunological protection and clinical outcomes. We have previously reported interim shorter-term (up to 3-months) comparisons between these two vaccines following receipt of a fourth vaccine dose [3,4]. We now report results from a longer-term (6 months) follow-up. This study was performed in a context where the Omicron (Phylogenetic Assignment of Named Global Outbreak (Pango) lineage designation: B.1.1.529) variant and its sub-variants (BA.1, BA.2, and BA.5) were dominant.

## Study setting and design

This was an open-label intervention study conducted among healthcare workers (HCW) at a large tertiary medical centre in Israel, nested within a larger observational cohort of HCWs from which the participants were recruited. The study period began on 27 December 2021, and we report on follow-up up to 24 July 2022. Individuals who received three doses of the Comirnaty vaccine at least 4 months prior, were not previously infected with severe acute respiratory syndrome coronavirus 2 (SARS-CoV-2), and had anti-receptor binding domain (RBD) immunoglobulin G (IgG) levels below 700 binding antibody units (BAU) during the 3 months prior, were assigned to receive either the Comirnaty vaccine (if enrolled on 27–28 December 2021) or the Spikevax vaccine (if enrolled on 5–6 January 2022). Aged-matched infection-naïve HCWs from the parent study, who had received three Comirnaty vaccine doses at least 4 months prior and had RBD IgG levels below 700 BAU during the 3 months prior, but who did not receive an additional vaccine dose as part of this study, were used as controls for the vaccine efficacy (VE) analysis. Follow-up of the vaccine recipients began on vaccination day and ended at the earlier of 181 days (6 months) or a positive SARS-CoV-2 test. For controls, follow-up began at the start of the study period and ended at the earliest of 181 days, a positive SARS-CoV-2 test, or receipt of a fourth vaccine dose outside the study.

Conduct of the study was previously reported in detail [3,4]. Participants underwent serological testing at predefined intervals, including at the day of recruitment and after 6 months. All participants were encouraged to test for SARS-CoV-2 infection using quantitative real-time PCR (qRT-PCR) or an antigen rapid diagnostic test (Ag-RDT) following any exposure to a person with SARS-CoV-2 or development of symptoms, and at least once a week. Individuals who were found to be infected were questioned about their symptoms and disease severity. Complete descriptions of the laboratory methods, the variables and the questionnaire used in this study are included in the Supplementary Methods. Analysis was performed using the R programming language, version 4.1.2 [5].

## Immunogenicity

In this analysis, the exposure was the type of vaccine received, and the outcomes were IgG levels (using SARS-CoV-2 IgG II Quant; Abbott, IL, US) and neutralising antibody levels (using SARS-CoV-2 pseudovirus neutralisation assay [6]). Geometric mean titres (GMT) of the antibody levels at baseline (day 0 of follow-up) and at 6 months post-vaccination, with their 95% confidence intervals (CI), were estimated from the crude observed values. The ratio between these two values was used to estimate the geometric mean fold rise.

Overall, 700 individuals were included in the study: 154 individuals in the Comirnaty arm, 120 in the Spikevax arm, and 426 in the control arm. The median age in the study population was 58 years (interquartile range: 46–67) and 70% (n = 490) of individuals were female (Supplementary Table S2; sex was collected as a binary variable). All individuals in the study had detectable neutralising antibodies at baseline and 6 months following vaccination. Immunological response, for both IgG levels and neutralising antibody titres, was mostly similar between the two vaccinee groups at 6-month follow-up, with perhaps a small advantage for the Spikevax vaccine (Figure A and B). At 6 months, IgG levels following Spikevax and Comirnaty doses were 1.58-fold (95% CI: 1.27–1.97) and 1.16-fold (95% CI: 0.98–1.37) higher than baseline, respectively, and neutralising antibody titres were 1.04-fold (95% CI: 0.74-1.45) and 0.75-fold (95% CI: 0.51-1.1) the level at baseline, respectively (Supplementary Table S3).

**Figure:**
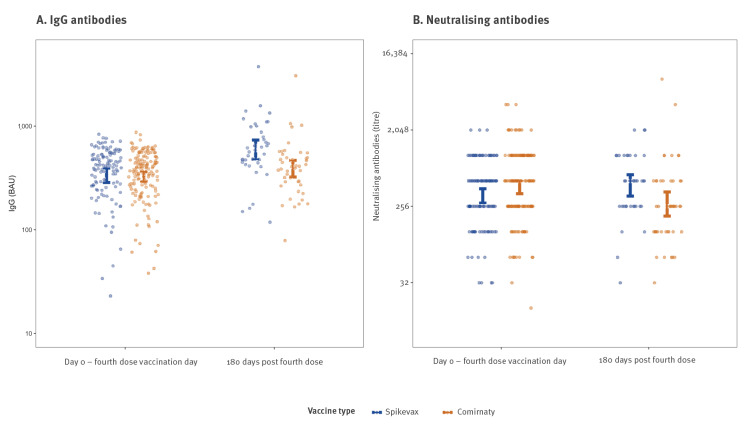
Estimated geometric mean titres (with 95% confidence interval) of (A) IgG and (B) neutralising antibodies, at baseline and 180 days post-vaccination, according to the vaccine received, Israel, 29 December 2021–6 January 2022 (n = 274)

BAU: binding antibody units.

## Vaccine efficacy

In the VE analysis, the exposure was the type of vaccine received, and the outcomes were SARS-CoV-2 infection (defined as a positive qRT-PCR or Ag-RDT test, or an abrupt increase in SARS-CoV-2 RBD IgG levels not related to vaccination) and substantial disease (defined as a SARS-CoV-2 infection that resulted in an individual spending 2 days or more in bed due to feeling unwell). VE for infection and substantial disease, over the entire study period, was estimated using a Cox proportional hazards model, adjusted for age and sex, such that a hazard ratio (HR) of 1 corresponds to no effect. Individuals with missing outcome data for substantial disease were dropped from the corresponding VE analysis.

By the end of the study, 70/120 (58.3%) and 89/154 (57.8%) of the Spikevax and Comirnaty vaccine recipients, respectively, contracted SARS-CoV-2, and 7/108 (6.5%) and 14/140 (10.0%), respectively, had substantial disease, though none required hospitalisation or medical attendance. VE against infection over the entire study period, compared with controls vaccinated with three doses at least 4 months prior, was similar and not statistically significant in both vaccine groups, with a hazard ratio (HR) of 0.86 (95% CI: 0.65–1.13) for Comirnaty and a HR of 0.82 (95% CI: 0.62–1.09) for Spikevax. VE against substantial disease was higher, with a non-significant advantage for the Spikevax vaccine; with a HR of 0.51 (95% CI: 0.27–0.95) for Comirnaty and a HR of 0.28 (95% CI: 0.13–0.62) for Spikevax (Supplementary Table S4).

## Discussion

Thirty days after the second and third doses, mRNA-based COVID-19 vaccines have been shown to elicit strong immune responses [6-8] and to be effective in reducing the incidence of infection and disease, as well as disease severity [9-11]. Over time, however, both the immunological response and VE were found to wane [12,13]. In early November 2021, the Omicron variant emerged [14], with, later in that month, appearance of sub-variants thereof. All of these variants demonstrated considerable vaccine escape, coinciding with a significant decrease in VE. Considering both waning of vaccine-induced immunity and the vaccine-escape capacity of Omicron and its sub-variants, many countries opted to offer ‘booster’ doses of vaccines. These doses were expected to restore immunity back to peak level and to reduce the probability of infection to that observed in the weeks following vaccination. Of the mRNA-based COVID-19 vaccines, both the Comirnaty and the Spikevax vaccines have been used to provide booster doses worldwide.

In this study, we observed that 6 months-post-fourth dose, antibody levels and cumulative VE do not differ between the two vaccines, with only a minor, non-significant advantage for Spikevax. We additionally observed that the immunological response to both vaccines waned in the months post-fourth dose to approach pre-booster levels at 6 months-post-fourth dose. Nearly 60% of the fourth vaccine dose recipients contracted SARS-CoV-2 within the 6-month follow-up. The cumulative efficacy of both vaccines for preventing infection was low, though somewhat better for preventing substantial disease.

The main limitation of our study is the small sample size, which results in wide confidence intervals and does not allow us to assess the less common more severe outcomes (e.g. hospitalisation and death). We note, however, that the existence of detectable neutralising antibody activity in all study participants suggests that some degree of protection from severe disease does exist. An additional limitation is that the study population is composed entirely of HCWs, raising the issue of generalisability. On the other hand, this HCW population does include volunteers, many older than retirement age. The main strength of our study is the intense follow-up of individuals in this HCW cohort, which makes outcome misclassification less likely.

Our findings suggest that choosing with which of the two vaccines to provide booster doses should depend mostly on availability. They further suggest that boosters with both vaccines are only effective for a short period in preventing infections by Omicron variants. While efficacy against substantial disease is significant, next-generation COVID-19 vaccines, tailored to new emerging variants, seem needed to provide improved protection against infection. Furthermore, recipients of the next-generation vaccines should be intensely monitored to detect waning and breakthrough infections, given the knowledge accumulated regarding COVID-19 vaccines so far.

## Supplementary Data

789000 Supplementary Material

## References

[1] Polack FP Thomas SJ Kitchin N Absalon J Gurtman A Lockhart S C4591001 Clinical Trial Group . Safety and Efficacy of the BNT162b2 mRNA Covid-19 Vaccine. N Engl J Med. 2020;383(27):2603-15. 10.1056/NEJMoa2034577 33301246 PMC7745181

[2] Baden LR El Sahly HM Essink B Kotloff K Frey S Novak R COVE Study Group . Efficacy and Safety of the mRNA-1273 SARS-CoV-2 Vaccine. N Engl J Med. 2021;384(5):403-16. 10.1056/NEJMoa2035389 33378609 PMC7787219

[3] Regev-Yochay G Gonen T Gilboa M Mandelboim M Indenbaum V Amit S Efficacy of a Fourth Dose of Covid-19 mRNA Vaccine against Omicron. N Engl J Med. 2022;386(14):1377-80. 10.1056/NEJMc2202542 35297591 PMC9006792

[4] Canetti M Barda N Gilboa M Indenbaum V Mandelboim M Gonen T Immune Response and Clinical Outcomes of BNT162b2 and mRNA1273 Fourth Dose COVID-19 Vaccines; Three Months Follow-up. Research Square 2022 Aug 11 10.21203/rs.3.rs-1946528/v1

[5] R Core Team. R: A Language and Environment for Statistical Computing. Vienna, Austria: R Foundation for Statistical Computing; 2020. Available from: https://www.R-project.org/

[6] Lustig Y Sapir E Regev-Yochay G Cohen C Fluss R Olmer L BNT162b2 COVID-19 vaccine and correlates of humoral immune responses and dynamics: a prospective, single-centre, longitudinal cohort study in health-care workers. Lancet Respir Med. 2021;9(9):999-1009. 10.1016/S2213-2600(21)00220-4 34224675 PMC8253545

[7] Lustig Y Gonen T Meltzer L Gilboa M Indenbaum V Cohen C Superior immunogenicity and effectiveness of the third compared to the second BNT162b2 vaccine dose. Nat Immunol. 2022;23(6):940-6. 10.1038/s41590-022-01212-3 35534723

[8] Chu L Vrbicky K Montefiori D Huang W Nestorova B Chang Y Immune response to SARS-CoV-2 after a booster of mRNA-1273: an open-label phase 2 trial. Nat Med. 2022;28(5):1042-9. 10.1038/s41591-022-01739-w 35241844 PMC9117133

[9] Haas EJ Angulo FJ McLaughlin JM Anis E Singer SR Khan F Impact and effectiveness of mRNA BNT162b2 vaccine against SARS-CoV-2 infections and COVID-19 cases, hospitalisations, and deaths following a nationwide vaccination campaign in Israel: an observational study using national surveillance data. Lancet. 2021;397(10287):1819-29. 10.1016/S0140-6736(21)00947-8 33964222 PMC8099315

[10] Barda N Dagan N Cohen C Hernán MA Lipsitch M Kohane IS Effectiveness of a third dose of the BNT162b2 mRNA COVID-19 vaccine for preventing severe outcomes in Israel: an observational study. Lancet. 2021;398(10316):2093-100. 10.1016/S0140-6736(21)02249-2 34756184 PMC8555967

[11] Tseng HF Ackerson BK Luo Y Sy LS Talarico CA Tian Y Effectiveness of mRNA-1273 against SARS-CoV-2 Omicron and Delta variants. Nat Med. 2022;28(5):1063-71. 10.1038/s41591-022-01753-y 35189624 PMC9117141

[12] Levin EG Lustig Y Cohen C Fluss R Indenbaum V Amit S Waning Immune Humoral Response to BNT162b2 Covid-19 Vaccine over 6 Months. N Engl J Med. 2021;385(24):e84. 10.1056/NEJMoa2114583 34614326 PMC8522797

[13] Ferdinands JM Rao S Dixon BE Mitchell PK DeSilva MB Irving SA Waning 2-Dose and 3-Dose Effectiveness of mRNA Vaccines Against COVID-19-Associated Emergency Department and Urgent Care Encounters and Hospitalizations Among Adults During Periods of Delta and Omicron Variant Predominance - VISION Network, 10 States, August 2021-January 2022. MMWR Morb Mortal Wkly Rep. 2022;71(7):255-63. 10.15585/mmwr.mm7107e2 35176007 PMC8853475

[14] Karim SSA Karim QA . Omicron SARS-CoV-2 variant: a new chapter in the COVID-19 pandemic. Lancet. 2021;398(10317):2126-8. 10.1016/S0140-6736(21)02758-6 34871545 PMC8640673

