# Supplementary material for: Comparing immunogenicity and efficacy of two different mRNA-based COVID-19 vaccines as a fourth dose; six-month follow-up, Israel, 27 December 2021 to 24 July 2022
Source: Euro Surveill. 2022 Sep 29;27(39):2200701. doi: 10.2807/1560-7917.ES.2022.27.39.2200701 (PMC9524052; doi:10.2807/1560-7917.ES.2022.27.39.2200701)
Supplement: Supplementary Material [file 2200701_SupplementaryMaterial.pdf]

Comparing Immunogenicity and Efficacy of Two Different mRNA-based COVID-19 Vaccines as a Fourth Dose; Six-Month Follow-up, Israel, 27 December 2021 to 24 July 2022

This supplementary material is hosted by *Eurosurveillance* as supporting information alongside the article "Comparing Immunogenicity and Efficacy of Two Different mRNA-based COVID-19 Vaccines as a Fourth Dose; Six-Month Follow-up, Israel, 27 December 2021 to 24 July 2022" on behalf of the authors, who remain responsible for the accuracy and appropriateness of the content. The same standards for ethics, copyright, attributions, and permissions as for the article apply. Supplements are not edited by *Eurosurveillance* and the journal is not responsible for the maintenance of any links or email addresses provided therein.

Table of Contents

Supplementary Methods S1 – Immunologic Methods....2

Supplementary Methods S2 – SARS-CoV-2 Cases....3

Supplementary Methods S3 – SARS-CoV-2 Symptoms and Disease Severity Questionnaire..4

Supplementary Table S1 – Variable Definitions....5

Supplementary Table S2 – Baseline Characteristics of the Study Population....6

Supplementary Table S3 – Immune Markers....7

Supplementary Table S4 – Vaccine Efficacy....8

Supplementary Methods S1 – Immunologic Methods

SARS-CoV-2 IgG II Quant (Abbott, IL, USA)

Samples were centrifuged at room temperature, at 4000g, for 4 minutes. Serum was tested for immunoglobulin G (IgG) antibodies against the severe acute respiratory syndrome coronavirus 2 (SARS-COV-2) spike receptor binding domain (RBD) using the commercial automatic chemiluminescent microparticle immunoassay (CMIA) SARS-CoV-2 IgG II Quant (Abbott, IL, USA) according to the manufacturer's instructions. The geometric mean titers (GMT) are presented in binding antibody units (BAU).

SARS-CoV-2 Pseudovirus (psSARS-2) neutralization assay

SARS-CoV-2 Pseudovirus (psSARS-2) neutralization assay was performed using a propagation-competent vesicular stomatitis virus spike and shown to be highly correlative to authentic SARS-CoV-2 virus micro-neutralization assay (1). Following titration, 100 focus forming units (FFU) of psSARS-2 were incubated with 2-fold serial dilution of heat inactivated (56°C for 30 min) tested sera. After incubation for 60 min at 37°C, virus/serum mixture was transferred to Vero E6 cells that have been grown to confluence in 96-well plates and incubated for 90 min at 37°C. After the addition of 1% methyl cellulose in dulbecco's modified eagle's medium (DMEM) with 2% of fetal bovine serum (FBS), plates were incubated for 24 hours, and 50% plaque reduction titer was calculated by counting green fluorescent foci using a fluorescence microscope (EVOS M5000, Invitrogen). Sera not capable of reducing viral replication by 50% at 1 to 16 dilution or below were considered non-neutralizing. For clear presentation, non-neutralizing samples were marked as a titer of 2.

Supplementary Methods S2 – SARS-CoV-2 Cases

Cases were defined as positive in one of three scenarios:

1. Positive SARS-CoV-2 quantitative real-time polymerase chain reaction (qRT-PCR) test. These tests were performed using the Allplex™-2019 nCoV (Seegene, S. Korea) platform.
2. Positive antigen rapid diagnostic test (Ag-RDT) test. These tests were performed using STANDARD Q COVID-19 Ag (SD BIOSENSOR, S. Korea).
3. an observation of an uncharacteristic increase in IgG levels, defined as >500 BAU or >1000 BAU in healthcare workers (HCW) with previous IgG results of <700 or >700, respectively.

Supplementary Methods S3 – SARS-CoV-2 Symptoms and Disease Severity Questionnaire

1. What was the highest Fever measurement in the past 24 hours?
   - Under 37
   - 37-37.4
   - 37.5-37.9
   - 38-38.9
   - 39-39.9
   - Over 40
2. What was the lowest saturation measured in the past 24 hours?
   - Above 96
   - Under 96
   - Not measured
3. Please mark all symptoms that you experienced in the past 24 hours:
   - Cough
   - Dyspnea
   - Myalgia
   - Throat pain
   - Headache
   - Chest pain
   - Fatigue
   - Rhinorrhea
   - Diarrhea
   - Anosmia
   - Other- please elaborate
   - I did not experience any symptom
4. How would you describe your functional status?
   - I am hospitalized
   - I cannot get out of bed
   - I feel unwell and spend most day in bed
   - I feel less well than usual
   - I feel well as usual

Supplementary Table S1 – Variable Definitions

Variable	Values	Definition
A. Exposure		
Vaccine type	BNT162b2/ mRNA1273	Vaccine type received upon enrollment
B. Immunological outcomes		
IgG	Numeric	Anti-RBD IgG GMT tested on days 0 and 180.
Neutralizing antibodies	Numeric	Neutralizing antibodies GMT on days 0 and 180.
C. Clinical outcomes		
SARS-CoV-2 infection	0/1	Positive SARS-CoV-2 qRT-PCR test, a positive Ag-RDT, or an uncharacteristic IgG increase after a decline from peak vaccination levels was already observed (>500 or >1000 from previous levels of <700 or >700, respectively)
SARS-CoV-2 substantial disease	0/1	Positive SARS-CoV-2 participant who spent two or more days mostly in bed due to feeling unwell
D. Covariates		
Age	Numeric	Participant's age upon enrollment
Sex	Female/ male	Participant's sex
BMI	Numeric	BMI calculated as weight/m^2 upon enrollment
Immunosuppression	0/1	Including organ transplant recipient, currently undergoing biological therapy or chemotherapy, treated with corticosteroids, underwent splenectomy, or diagnosed with HIV
Number of comorbidities	0, 1, +2	Including hypertension, dyslipidemia, autoimmune disease, diabetes, heart disease, lung disease, coagulation disorder, liver disease, and kidney disease

Abbreviations: IgG- anti-RBD immunoglobulin G, GMT- geometric mean titers, SARS-CoV-2- severe acute respiratory syndrome coronavirus 2, qRT-PCR- quantitative real-time polymerase chain reaction, Ag-RDT- antigen rapid diagnostic test, BMI- body mass index, HIV- human immunodeficiency virus.

Supplementary Table S2 – Baseline Characteristics of the Study Population

Characteristics of the study population following application of all eligibility criteria. IQR: Interquartile Range.

Variable	Entire Population N=700	Control N=426	mRNA1273 N=120	BNT162b2 N=154
Age, Median (IQR)	58 (46, 67)	58 (46, 67)	56 (46, 64)	61 (47, 70)
Sex, N (%)				
Female	490 (70%)	318 (75%)	81 (68%)	91 (59%)
Male	210 (30%)	108 (25%)	39 (32%)	63 (41%)
Professional Role, N (%)				
Physicians	-	-	35 (29%)	41 (27%)
Nurses	-	-	21 (18%)	34 (22%)
Paramedical personnel	-	-	35 (29%)	34 (22%)
Administrative personnel	-	-	26 (22%)	43 (28%)
Other	-	-	3 (2.5%)	2 (1.3%)

Supplementary Table S3 – Immune Markers

The geometric mean titer for each vaccination type and measurement at baseline and at 6-month follow-up and the geometric mean fold rise when comparing values at 6 months and at baseline. BAU: Binding Antibody Units, GMT: Geometric Mean Titer, GMFR: Geometric Mean Fold Rise.

		Baseline		Six Months		
Measurement	Vaccination Type	N	GMT	N	GMT	GMFR*
IgG (BAU)	mRNA1273	119	336 (286-394)	40	593 (480-734)	1.58 (1.27-1.97)
	BNT162b2	154	326 (293-363)	44	388 (323-467)	1.16 (0.98-1.37)
Neutralizing Antibodies (Titer)	mRNA1273	118	339 (279-413)	39	452 (338-605)	1.04 (0.74-1.45)
	BNT162b2	154	430 (361-512)	45	272 (196-378)	0.75 (0.51-1.1)

* GMFR values were estimated using only those individuals with measurements at both baseline and six months.

Supplementary Table S4 – Vaccine Efficacy

Number of individuals analyzed, total follow-up days contributed, number of cases, the incidence rate, and the hazard ratio (estimated using a Cox proportional hazards model adjusted for age and sex with the controls as baseline) over the six-month follow-up period. Only individuals with non-missing data regarding infection symptoms were included in the analysis for substantial disease. CI: Confidence Interval.

Outcome		N	Follow-up Days Contributed	Positive Cases	Incidence Rate per 1000 days	Hazard Ratio (95% CI)
Infection	Controls	426	22,342	150	6.71	Reference
	mRNA1273	120	13,835	70	5.06	0.82 (0.62-1.09)
	BNT162b2	154	17,849	89	4.99	0.86 (0.65-1.13)
Substantial Disease	Controls	393	19,051	48	2.52	Reference
	mRNA1273	108	12,311	7	0.57	0.28 (0.13-0.62)
	BNT162b2	140	16,065	14	0.87	0.51 (0.27-0.95)

References

1) Lustig Y, Sapir E, Regev-Yochay G, et al. BNT162b2 COVID-19 vaccine and correlates of humoral immune responses and dynamics: a prospective, single-centre, longitudinal cohort study in health-care workers. Lancet Respir Med 2021;9(9):999–1009.
